# A multicenter prospective phase II randomized trial of epirubicin/vinorelbine versus pegylated liposomal doxorubicin/vinorelbine as first-line treatment in advanced breast cancer. A GOIM study

**DOI:** 10.1186/1756-9966-30-39

**Published:** 2011-04-12

**Authors:** Patrizia Vici, Giuseppe Colucci, Francesco Giotta, Domenico Sergi, Gianfranco Filippelli, Pasquale Perri, Claudio Botti, Enrico Vizza, Armando Carpino, Laura Pizzuti, Agnese Latorre, Diana Giannarelli, Massimo Lopez, Luigi Di Lauro

**Affiliations:** 1 Division of Medical Oncology B, Regina Elena National Cancer Institute, Rome, Italy; 2 Department of Medical and Experimental Oncology, Oncologic Institute, Bari, Italy; 3 Division of Medical Oncology, S. Francesco Hospital, Paola, Italy; 4 Division of Surgery A, Regina Elena National Cancer Institute, Rome, Italy; 5 Division of Gynecological Oncology, Regina Elena National Cancer Institute, Rome, Italy; 6 Cardiologic Unit, Regina Elena National Cancer Institute, Rome, Italy; 7 Biostatistics Unit, Regina Elena National Cancer Institute, Rome, Italy

## Abstract

**Background:**

To evaluate activity and tolerability of two anthracycline-containing regimens as first-line treatment for anthracycline-naïve relapsed breast cancer patients.

**Methods:**

Patients with relapsed breast cancer not previously treated with adjuvant anthracyclines were randomly assigned to epirubicin/vinorelbine (arm A: EPI/VNB, EPI 90 mg/m^2 ^on day 1, VNB 25 mg/m^2 ^on days 1,5 plus G-CSF subcutaneously on days 7-12, with cycles repeated every 21 days), or to pegylated liposomal doxorubicin/VNB (arm B: PLD/VNB, PLD 40 mg/m^2 ^on day 1, VNB 30 mg/m^2 ^on days 1, 15, with cycles repeated every 4 weeks). Primary objective was to evaluate the efficacy of the two regimens in terms of response rate, secondarily toxicity, progression free survival and overall survival.

**Results:**

One hundred and four patients have been enrolled (arm A 54, arm B 50): characteristics were well balanced between the 2 arms. Responses were as follows: arm A, 3 (5.6%) CR, 20 (37%) PR, (ORR 42.6%, 95%CI 29.3%-55.9%); arm B, 8 (16%) CR, 18 (36%) PR, (ORR 52%, 95%CI 38.2%-65.8%). Median progression free survival was 10.7 months in arm A (95% CI, 8.7-12.6), and 8.8 months in arm B (95% CI, 7.1-10.5). Median overall survival was 34.6 months in arm A (95%CI, 19.5-49.8) and 24.8 months in arm B (95%CI, 15.7-33.9). As toxicity concerns, both treatment regimens were well tolerated; myelosuppression was the dose-limiting toxicity, with G3-4 neutropenia occurring in 18.5% and 22% of the patients of arm A and B, respectively. No relevant differences in main toxic effects have been observed between the two arms, except for alopecia, more common in arm A, and cutaneous toxicity, observed only in arm B. No clinical congestive heart failures have been observed, one case of tachyarrhythmia was reported after the last EPI/VNB cycle, and two reversible ≥ 20% LVEF decreases have been observed in arm A.

**Conclusions:**

Both anthracycline- containing regimens evaluated in the present study seem to be active and with a satisfactory tolerability in anthracycline-naïve relapsed breast cancer patients.

## Background

Anthracyclines are among the most active drugs in advanced breast cancer, with response rates as single agents of approximately 30% to 50%, and anthracycline-based regimens have been shown to determine significant advantages in response rate and progression free survival over non- anthracycline-containing regimens [1,2]. The potential benefit of conventional anthracyclines, mainly doxorubicin, is limited by the risk of cardiac dysfunction, clearly related to cumulative dose, and as a result it might be necessary to withdraw treatment or to avoid re-treatment even in potential responders patients.

To minimize toxic effects, doxorubicin has often been replaced by epirubicin (EPI), known to be as active as the parent compound and with lower toxicity, particularly cardiac toxicity [3-6]. As dose-response concerns, higher EPI doses, both as single agent and in combination regimens, seem to be more efficacious than lower doses [7-10].

Vinorelbine (VNB) has established activity as single-agent in breast cancer, both as first-line and salvage treatment [11,12], and its good tolerance profile makes it an excellent candidate for combination regimens, so it was a logical step to combine VNB with anthracyclines, and the combination with doxorubicin yielded a 74% of response rate, a median duration of response of 12 months and a median survival of 27.5 months as first-line treatment [13]. Other trials employing this combinations confirmed positive results [14-16]. Preliminary results of a randomized phase III trial comparing VNB 25 mg/m^2 ^on days 1,8 in combination with EPI 90 mg/m^2^, with EPI as single agent, showed a trend for higher response rate (50% vs 42%) and a significantly longer progression free survival (10.1 vs 8.2 months) for the combination arm [17]. An our previous clinical study with EPI/VNB combination as first-line treatment showed a very high activity (70.6% of response rate) and acceptable toxicity [18]; another our experience testing the sequential administration of docetaxel for 4 cycles followed by 4 cycles of EPI/VNB as first-line treatment for advanced disease, confirmed activity and tolerability of the regimen [19].

Incapsulating drugs in liposomes determine improvement of solubility and stability of the drug, and prevent a rapid degradation; moreover, specific toxicities are potentially lowered and the efficacy increased, achieving a higher therapeutic index [20]. Liposomal anthracyclines exhibit efficacies comparable with those of conventional anthracyclines, but with better safety profiles [21-24]. In particular, data from retrospective analyses showed that liposomal anthracyclines significant reduced the risk of cardiotoxicity compared with conventional anthracyclines [25]. Phase III trials comparing pegylated liposomal doxorubicin (PLD) with conventional anthracyclines confirmed similar efficacy and lower toxicity than doxorubicin [24,26], and results of several studies have shown that PLD is effective in combination with other drugs including taxanes, cyclophosphamide, gemcitabine [27]. As cardiotoxicity concerns, in a retrospective analysis a low incidence of cardiac side effects were reported, even at cumulative doses higher than 500 mg/m^2 ^[28]. The combination of PLD with VNB was investigated in anthracycline pretreated patients, with promising results and manageable toxicity [29,30], but at the time we design the present study no information about its first-line use in comparison with a conventional anthracycline-containing regimen were available, so we carried out a prospective multicenter phase II randomized trial of EPI/VNB versus PLD/VNB as first-line treatment for advanced disease in patients not previously treated with adjuvant anthracyclines.

## Patients and Methods

### Patient selection

Patients with histologically proven advanced breast cancer not previously treated with adjuvant anthracyclines were enrolled. Eligibility criteria included a life expectancy > 3 months, 18 to 75 years of age, WHO performance status ≤ 3, measurable/assessable disease, adequate bone marrow (absolute neutrophil count ≥1,500, platelet count ≥ 100,000, haemoglobin ≥ 11 g/dL), renal and liver function (total bilirubin and creatinine <1.25 times the upper normal limits), and a normal cardiac function (left ventricular ejection fraction LVEF ≥ 50% by echocardiography). Patients were excluded from the study if they had active cardiac diseases or significant arrhythmias, pre-existent neuropathy, or had received prior chemotherapy treatment for advanced disease, prior exposure to anthracyclines and or vinorelbine, or if they had prior or concomitant malignant disease, except appropriately treated basal cell carcinoma of the skin or in situ carcinoma of the cervix. Previous adjuvant chemotherapy with cyclophosphamide, methotrexate, fluorouracil or similar regimens were allowed, but an interval of two months should have elapsed; hormonal adjuvant treatment and radiotherapy must have been discontinued for at least 4 weeks before study entry. The protocol was approved by the ethical committees of each participant centers, and was carried out according to Helsinki declaration and in accordance with the International Conference on Harmonization Good Clinical Practice guidelines.

### Treatment

Patients were centrally assigned according to a computer generated random list to receive either (arm A) EPI 90 mg/m^2 ^i.v. on day 1 plus VNB 25 mg/m^2 ^i.v on days 1 and 5, with granulocyte colony-stimulating factor (G-CSF) subcutaneously on days 7-12 of each cycle, or (arm B) PLD 40 mg/m^2 ^i.v. on day 1, plus VNB 30 mg/m^2 ^on days 1 and 15. Cycles were repeated every 21 days in arm A, and every 28 days in arm B, for a maximum of 8 cycles. Treatment was continued until disease progression, severe toxicity, patient refusal. Antiemetic treatment consisted of an antiserotonin agent plus desamethasone in a 15 min infusion before starting chemotherapy. Treatment was postponed by a maximum of 2 weeks if the absolute neutrophil count was less than 1,500**/**μL or the platelet count was less than 100,000**/**μL. A 25% drugs dose-reduction was planned in case of grade 4 neutropenic fever, as well as in case of grade 3 mucositis or neurotoxicity. G-CSF was administered in arm B in case of grade 4 neutropenic fever, and prophylactively in the subsequent cycles. Treatment was discontinued in case of grade 4 neurotoxicity, mucositis, palmar plantar erythrodisesthesia (PPE), treatment delay longer than 2 weeks, or in case of cardiotoxicity, defined as LVEF decrease ≥ 20% from baseline, or ≥10% but with a value below 50%, or any symptoms of congestive heart failure or arrhythmias even in absence of LVEF decrease. Hematologic assessment was done on days 1 and 12 of every cycle in arm A, and on days 1 and 14 in arm B, and whenever useful at discretion of investigator.

### Pretreatment and Follow Up Studies

Pretreatment investigations included complete blood count and chemistry, chest x-ray, bone scan, CT abdomen, LVEF evaluation by echocardiography, and other site-specific imaging as appropriate. Echocardiography with LVEF evaluation had to be performed every 3 cycles, or whenever indicated at discretion of investigator; during the follow-up LVEF had to be determined every 6 months.

### Evaluation of Response and Toxicity

Tumor assessment was performed every 3 cycles, or whenever appropriate, and responses were evaluated according to RECIST criteria [31]. Progression free survival (PFS) was calculated starting from the date of randomization to the date of disease progression, refusal or death from any cause; overall survival (OS) was calculated starting from the date of randomization to the date of death or last follow up evaluation. Toxicity was assessed in each cycle according to National Cancer Institute Common Toxicity Criteria (version 3.0).

### Statistical Methods

The primary objective of the study was to evaluate the overall response rate of the two regimens, secondarily toxicity, progression free survival and overall survival.

The study was designed as a phase II trial with a random assignement to a calibration arm A and to an experimental arm B. The sample size for arm B was calculated according to the design described by A'Hern [32]. A sample size of 53 patients was considered sufficient to give a 90% probability of rejecting a baseline response rate of 35% with an exact 5% one-sided significance test when the true response rate was 55%. The drug regimen should have been considered for further studies if at least 25 responses were observed. The calibration arm had the same sample size. No formal comparison was planned. The objective response rate have been reported with its 95% confidence interval. All patients enrolled were considered in the intention-to-treat population (ITT). This population have been evaluated for the efficacy analysis, which was performed also on evaluable patients. Subjects who assumed at least one dose of drug have been considered as denominator in the safety analysis.

The time to event analysis was performed according the Kaplan-Meier method.

## Results

### Patients Characteristics

From March 2003 to November 2005, a total of 104 patients were enrolled from 4 oncologic centers of the GOIM (Gruppo Oncologico Italia Meridionale), with 54 patients randomized to arm A (EPI/VNB) and 50 patients to arm B (PLD/VNB). All randomized patients have been evaluated for activity and toxicity according to ITT analysis. Patient characteristics are listed in Table 1. None of the patients have received any chemotherapy for advanced disease; 20 patients in arm A and 21 patients in arm B had received adjuvant chemotherapy, not including anthracyclines or vinka alcaloids; 35 and 30 patients had received previous adjuvant hormonal therapy, and 10 and 11 patients had received endocrine treatment for advanced disease in arm A and B, respectively. Median age was 63 and 61 years, 10 and 9 patients were premenopausal, 44 and 41 postmenopausal in arm A and B, respectively; dominant site of disease was soft tissue in 3 (5.6%) and 9 (18.0%), bone in 11 (20.4%) and 9 (18.0%), viscera in 40 (74.0%) and 32 (64.0%) patients in arm A and B, respectively. Hormonal receptors were positive (ER and/or PgR) in 39 and 32 patients, negative in 13 and 15 patients, unknown in 2 and 3 patients in the two arms, respectively. Her-2, retrospectively evaluated in 35 and 38 patients in arm A and B, was overexpressed or amplified in 8 patients in each arm (14.8% and 16%, respectively). The median number of chemotherapy cycles administered was 6 in both arms (range, 1 to 8 in both arms).

**Table 1:** Patient and tumor characteristics

Characteristics	Arm A(EV) = 54		Arm B(PLD/V) = 50	
	
	**No**.	%	**No**.	%
Age, years				
Median	63		61	
Range	27-70		35-69	

Pre/Postmenopausal	10/44		9/41	

Median ECOGPS (range)	1(0-3)		1(0-3)	

*HR status				
Positive	39	72.2	32	64.0
Negative	13	24.1	15	30.0
Unknown	2	3.7	3	6.0

HER-2 status				
Positive				
Negative				
Unknown				

Prior adjuvant chemotherapy**	20	37	21	42

Prior hormonal therapy				
Adjuvant	35	64.8	30	60
Advanced	10	18.5	11	22

Disease free-interval (years)				
< 1	10		11	
1-5	30		28	
>5	14		11	

Dominant disease site				
Viscera	40	74.0	32	64.0
Bone	11	20.4	9	18.0
Soft tissue	3	5.6	9	18.0

Number of disease site				
1	23	42.6	23	46.0
2	23	42.6	18	36.0
≥ 3	8	42.6	9	18.0

* HR: hormonal receptor status

** not including anthracyclines or vinka alkaloids

EV: epirubicin/vinorelbine; PLD/V: pegylated liposomal doxorubicin/vinorelbine

### Efficacy

**Figure 1:**
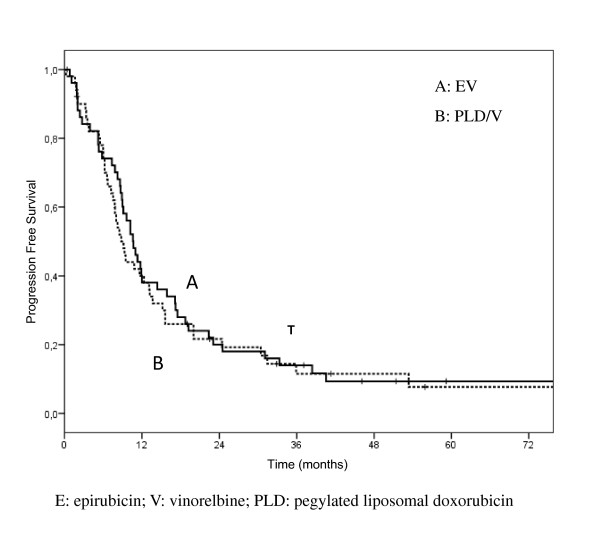
**Progression Free Survival**.

**Figure 2:**
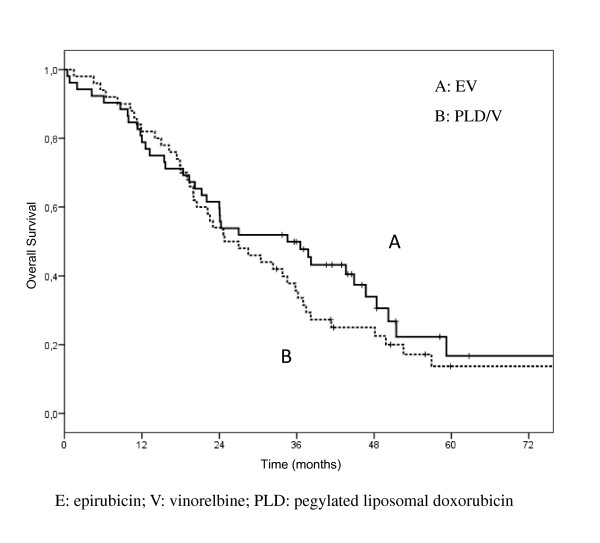
**Overall Survival**.

According to an intent to treat analysis, among 54 patients enrolled in arm A, there were 3 complete response (5.6%) and 20 partial responses (37%), for an overall response rate of 42.6% (95% CI, 29.3-55.9); disease remained stable in 19 (35.2%), and progressive disease was observed in 6 (11.1%) patients. Among 50 patients enrolled in arm B, there were 8 complete responses (16%) and 18 partial responses (36%), for an overall response rate of 52% (95% CI, 38.2-65.8); disease remained stable in 12 (24%), and disease progression occurred in 9 (18%) patients (Table 2a). Six patients of arm A and 3 patients of arm B were not evaluable for response (4 refusal, 5 lost to follow up). Objective response rates in 48 and 47 evaluable patients were 47.9% (95% CI, 33.9-61.9), and 55.3% (95% CI, 41.1-69.4) in the arm A and B, respectively (Table 2b). Disease control (CRs + PRs + NC) was 87.5% in arm A and 80.8% in arm B, respectively. Responses according to disease sites in evaluable patients are reported in details on Table 2c, and were as follows: arm A/B, soft tissue 66.6%/77.7%; bone 33.3%/37.5%; viscera 50%/53.3%. No relevant differences in response rate was observed according to hormonal receptor status, evidencing only a trend of higher response in receptor negative tumors in both arms (53.6% vs 45.7%, arm A; 60% and 53.1% arm B). No differences in response rates have been observed by Her-2 status in both arms, but numbers are very small: arm A Her-2 neg 54%, Her-2 pos 42.8%; arm B Her-2 neg 64%, Her-2 pos 50%. Median time to response was 2 months in both arms (range, 1 to 4 months). Median progression free survival (Figure 1) was 10.7 months in arm A (95% CI, 8.7-12.6), and 8.8 months in arm B (95% CI 7.1-10.5), median overall survival (Figure 2) was 34.6 months in arm A (95%CI, 19.5-49.8) and 24.8 months in arm B (95% CI, 15.7-33.9).

**Table 2:** Objective responses

2a. ITT on all enrolled patients						
	Arm A (EV) (54)			Arm B (PLD/V) (50)		
				
	No.	%		No.	%	
CR	3	5.6	42.6%	8	16.0	52.0%
PR	20	37.0	42.6%	18	36.0	52.0%
NC	19	35.2		12	24.0	
PD	6	11.1		9	18.0	
						
**2b. On evaluable patients**						
	Arm A (EV) (54)			Arm B (PLD/V) (47)		
				
	No.	%		No.	%	

CR	3	6.3	47.9%	8	17.0	55.3%
PR	20	41.6	47.9%	18	38.7	55.3%
NC	19	39.6		12	25.5	
PD	6	12.5		9	19.2	
						
**2c. Overall response rates according to disease sites in evaluable patients (%)**						
	Arm A (EV) (48)			Arm B (PLD/V) (47)		

Soft tissue	66.6			77.7		
Bone	33.3			37.5		
Viscera	50.			53.3		

Abbreviations: EV = epirubicin, vinorelbine; PLD/V = pegylated liposomal doxorubicin/vinorelbine; ITT = intent to treat; CR = complete response; PR = partial response; NC = no change; PD = progressive disease

### Toxicity

Table 3 summarizes treatment-related main toxicities. Overall, both treatment regimens were well tolerated. The dose-limiting toxicity was, as expected, myelosuppression, with G3-4 neutropenia occurring in 18.5% and 22% of the patients of arm A and B, respectively, with grade 3-4 neutropenic fever observed in 3 (5.5%) patients of arm A, and in 2 patients (4.0%) of arm B, in whom the administration of G-CSF was required. A 25% EPI/VNB dose-reduction was required in 7% of the patients, whereas a 25% PLD/VNB dose-reduction was required in 2 (4%) patients. Grade 3 thrombocytopenia was encountered only in one patient in arm A. Grade 3 alopecia was universal in arm A, whereas in arm B it was of grade 3 only in 50% of the patients. Mild (G1-2) nausea and vomiting was encountered in 46.3%/44.0% of the patients in the two arms, respectively. Grade 3 mucositis was observed in 7.4% and 12% of the patients in arm A and B, respectively. Reversible AST/ALT elevation was reported in 2 patients in both arms, and mild and transient peripheral neurotoxicity was observed in 8 and 7 patients in arm A and B, respectively, while it was of grade 3 in 1 patients in both arms. Grade 3 PPE or cutaneous toxicity was observed in 3 (6%) patients of arm B, usually related to treatment duration, and prompted to treatment discontinuation in 1 patient after 4 cycles. As cardiotoxicity concerns, no cases of congestive heart failure have been observed in the two arms. A transient and asymptomatic ≥ 20% LVEF decrease was encountered in 2 patients (3.7%) in arm A, and this prompted to treatment discontinuation after 5^th^, and 6th cycle; complete LVEF recovery was observed in two months. One case of transient and reversible supraventricular tachyarrhythmia was observed in arm A, during the last EPI infusion. The median cumulative delivered EPI dose was 540 mg/m^2 ^(range, 90 to 720 mg/m^2^); the median cumulative delivered PLD dose was 240 mg/m^2 ^(range, 40 to 320 mg/m^2^). No toxic deaths have been observed in the two arms.

**Table 3:** Grade 3-4 NCI-CTC toxicities in 104 enrolled patients

	Arm A (EV = 54)		Arm B (PLD/V = 50)	
	
	**No**.	%	**No**.	%
Anemia	5	9.2	4	8
Neutropenia	10	18.5	11	22
Thrombocytopenia	1	1.8	-	-
Febrile neutropenia	3	5.5	1	2.0
Hepatotoxicity	2	3.7	2	4.0
Mucositis	4	7.4	6	12
PPE/skin	-	-	3	6
Alopecia	54	100	25	50
Neurologic	1	1.8	1	2.0
Cardiac	2	3.7	-	-

Abbreviations: NCI-CTC: National Cancer Institute Common Toxicity Criteria; PPE: palmoplantar erithrodysthesia; EV: epirubicin/vinorelbine; PLD/V: pegylated liposomal doxorubicin/vinorelbine

## Discussion

Currently, there is no "gold standard" therapy for metastatic breast cancer, and treatment decisions must be based on patient and tumor characteristics, and on prior treatments. In the scenario of patients presenting with advanced disease, still exists a subgroup who have received only endocrine adjuvant therapy, or adjuvant chemotherapy with CMF or CMF-like regimens and, less frequently, there is a small cohort treated with adjuvant taxanes-based or other anthracycline-free regimens; moreover, there are also anthracycline pretreated patients with a very long free-interval, to be considered still anthracycline sensitive. In all these patient cohorts there is still the option to employ an anthracycline-based regimen as first-line treatment for advanced disease, mostly in hormonal receptor and/or Her-2 negative tumors, where a "targeted" therapy is not available.

The results of the present study confirm the activity of both anthracycline-based chemotherapy regimens for anthracycline-naïve advanced breast cancer patients, even if lower than expected. Response rate, progression free survival and overall survival observed in experimental arm B were comparable to those obtained in the "calibration" EPI/VNB arm. As toxicity concerns, both regimens were tolerable, with a higher incidence of febrile neutropenia and G3 alopecia in arm A, and of grade 3 mucositis and cutaneous toxicity in arm B. As cardiotoxicity concerns, the relatively low cumulative EPI dose delivered (≤ 720 mg/m^2^) did not allow to evidence significant clinical cardiotoxicity in the arm A, with only one case of arrhythmia, and a transient and asymptomatic in LVEF decrease occurring in 2 patients (3.7%), leading to a discontinuation of chemotherapy after 5 and 6 cycles, and with a complete recovery within two months.

Analyzing literature data, the EPI/VNB regimen is among the active, non-taxane, anthracycline-containing combinations for breast cancer treatment, as confirmed by definite results of the Scandinavian Breast Trial Group [33], and other trials [18], but some instances of clinical cardiac toxicity in terms of congestive heart failure or cardiomyopathy have been reported, with an incidence of asymptomatic LVEF decrease ranging from 20%-30% [33,34], so there is an urgent need of introduce new active and safer regimens for anthracycline-sensitive breast cancer patients, and a recent metanalysis showed a significant lower rate of both clinical and subclinical heart failure in patients treated with liposomal anthracyclines, compared with conventional doxorubicin [35].

A number of phase II trials have recently evaluated PLD in combination regimens with cyclophosphamide, paclitaxel, docetaxel, gemcitabine, VNB, and with biological agent such as trastuzumab or lapatinib, with response rates ranging from 31% to 75%, frequently occurring even in anthracycline pretreated patients [36], and with negligible cardiotoxicity. In details, the PLD/VNB combination was recently employed in two phase II trials in heavily pretreated patients, yielding a response rate of 36% and 39%, without any cardiac toxicity [37,38]. Two more recent reports with PLD/VNB combination as first-line treatment in elderly patients confirmed the good overall clinical response rate (36% and 50%, respectively), and **t**he high tolerability of the regimen [39,40] suggesting, due to the safety profile of the combination, the employment also in such "frail" patient population.

An increasingly pertinent question in patients relapsing following adjuvant anthracyclines is whether there is a role for anthracycline rechallenge in those with a long free-interval. As a result of a high cardiac risk associated with increasing cumulative anthracycline dose, patients are often denied re-treatment in advanced setting; the choice of a liposomal anthracycline allows the possibility of re-treating an anthracycline-responsive disease without substantially increasing the cardiac risk [36]; this option should not be excluded in fact, and some evidences come from a recent report on first- line chemotherapy selection in adjuvant anthracycline-pretreated patients, where no differences have been found between CMF-based and anthracycline-containing regimens for their impact on the outcome of first-line anthracycline treatment [41]. By this point of view, even if our results are in anthracycline-naïve patients, the activity and the low toxicity profile observed suggest that the choice of a liposomal formulation can offer the chance of a more tolerable regimen maintaing conventional anthracyclines efficacy.

The results of the present trial indicated both EPI/VNB and PLD/VNB as two reasonable choices as first-line treatment for women with relapsed breast cancer not previously treated with adjuvant anthracyclines; since advanced breast cancer is still an incurable disease, the goals of treatments are symptoms palliation with minimal toxicity, and survival prolongation, possibly with regimens active against cancer but also preserving patient's quality of life; in this context, our results are encouraging, confirming the feasibility and efficacy of two anthracycline-containing regimens and, particularly, of a regimen devoided of cardiac toxicity and of other severe side effects, such as PLD/VNB; the choice of this combination could offer a better quality of life and, hopefully, a better outcome to metastatic breast cancer patients.

## Conclusions

Both anthracycline-based regimens evaluated as first-line treatment in advanced breast cancer patients not previously treated with anthracyclines seems to be active and well tolerated, and can be considered as a reasonable choice in this subset of patients

## Competing interests

The authors declare that they have no competing interests.

## Authors' contributions

PV, GC, ML designed the study; FG, DS, GF, PP, CB, EV, AC, LP, AL, LDL collected and assembled the data, DG performed the statistical analysis, PV and LDL wrote the manuscript. All authors read and approved the final manuscript.
